# Computed tomography semi-automated lung volume quantification in SARS-CoV-2-related pneumonia

**DOI:** 10.1007/s00330-020-07271-0

**Published:** 2020-10-30

**Authors:** Davide Ippolito, Maria Ragusi, Davide Gandola, Cesare Maino, Anna Pecorelli, Simone Terrani, Marta Peroni, Teresa Giandola, Marco Porta, Cammillo Talei Franzesi, Sandro Sironi

**Affiliations:** 1 Department of Diagnostic Radiology, San Gerardo Hospital, University of Milano-Bicocca, Via Pergolesi 33, Monza, MB 20900 Italy; 2 School of Medicine, University of Milano-Bicocca, Via Cadore 48, Monza, MB 20900 Italy; 3 Philips Healthcare, Viale Sacra 235, Milan, MI 20126 Italy; 4 Department of Diagnostic Radiology, H Papa Giovanni XXIII, Piazza OMS 1, Bergamo, BG 24127 Italy

**Keywords:** Artificial intelligence, Computed tomography, X-ray, Lung volume measurements, Infection, coronavirus, Pneumonia

## Abstract

**Objectives:**

To evaluate a semi-automated segmentation and ventilated lung quantification on chest computed tomography (CT) to assess lung involvement in patients affected by SARS-CoV-2. Results were compared with clinical and functional parameters and outcomes.

**Methods:**

All images underwent quantitative analyses with a dedicated workstation using a semi-automatic lung segmentation software to compute ventilated lung volume (VLV), Ground-glass opacity (GGO) volume (GGO-V), and consolidation volume (CONS-V) as absolute volume and as a percentage of total lung volume (TLV). The ratio between CONS-V, GGO-V, and VLV (CONS-V/VLV and GGO-V/VLV, respectively), TLV (CONS-V/TLV, GGO-V/TLV, and GGO-V + CONS-V/TLV respectively), and the ratio between VLV and TLV (VLV/TLV) were calculated.

**Results:**

A total of 108 patients were enrolled. GGO-V/TLV significantly correlated with WBC (*r* = 0.369), neutrophils (*r* = 0.446), platelets (*r* = 0.182), CRP (*r* = 0.190), PaCO_2_ (*r* = 0.176), HCO_3_^−^ (*r* = 0.284), and PaO2/FiO2* (P*/*F*) values (*r* = − 0.344). CONS-V/TLV significantly correlated with WBC (*r* = 0.294), neutrophils (*r* = 0.300), lymphocytes (*r* = −0.225), CRP (*r* = 0.306), PaCO_2_ (*r* = 0.227), pH (r = 0.162), HCO_3_^−^ (*r* = 0.394), and *P*/*F* (*r* = − 0.419) values. Statistically significant differences between CONS-V, GGO-V, GGO-V/TLV, CONS-V/TLV, GGO-V/VLV, CONS-V/VLV, GGO-V + CONS-V/TLV, VLV/TLV, CT score, and invasive ventilation by ET were found (all *p* < 0.05).

**Conclusion:**

The use of quantitative semi-automated algorithm for lung CT elaboration effectively correlates the severity of SARS-CoV-2-related pneumonia with laboratory parameters and the need for invasive ventilation.

**Key Points:**

*• Pathological lung volumes, expressed both as GGO-V and as CONS-V, can be considered a useful tool in SARS-CoV-2-related pneumonia.*

*• All lung volumes, expressed themselves and as ratio with TLV and VLV, correlate with laboratory data, in particular C-reactive protein and white blood cell count.*

*• All lung volumes correlate with patient’s outcome, in particular concerning invasive ventilation.*

## Introduction

The novel coronavirus disease (SARS-CoV-2) has been declared a global pandemic by the World Health Organization on March 11, 2020. The disease rapidly spread in Northern Italy, with a special focus in Lombardy, with 82,904 confirmed cases and 15,118 deaths, as of May 12, 2020. The clinical spectrum of SARS-CoV-2 is wide: while the majority of infected individuals experience only a mild or subclinical illness, approximately 16 to 26% of hospitalized patients worsen, developing severe pneumonia, acute respiratory distress syndrome (ARDS), and multiple organ failure (MOF) that can ultimately lead to intensive care or death [1].

According to the WHO guidelines, real-time reverse-transcription polymerase chain reaction (RT-PCR) assay for SARS-CoV-2 diagnosis is the reference standard in daily practice [2]. Even if recently developed RT-PCR tests showed higher specificity and sensitivity compared with the previous ones, several studies published in literature [3] addressed the importance of chest computed tomography (CT) in patients suspected for SARS-CoV-2, considering its less time-consuming procedure, especially in patients with negative RT-PCR.

Early radiologic investigations consistently reported that typical SARS-CoV-2-related pneumonia CT findings are bilateral ground-glass opacities (GGOs) and consolidation with a peripheral and posterior lung distribution [4].

The proper assessment of disease severity, identifying the need for intensive care admission, may lead to the appropriate management, in particular addressing medical therapy and, eventually, establishing the need for invasive ventilation. In fact, since clinical evaluation may be misleading and patients may develop the so-called “silent hypoxemia” [5], the use of chest CT can be considered a rational, quantitative tool to evaluate residual and functioning lung volume and, ultimately, help to predict worsening and need for intensive care.

In the past years, CT lung volumetry has been employed to evaluate chronic obstructive pulmonary disease (COPD) along with pulmonary function tests [6–13].

Post-processing software, that can semi-automatically differentiate pulmonary tissue, in particular airways, emphysema, parenchymal thickening, fibrosis, and normal lung, plays a key role in this evaluation. As GGOs in SARS-CoV-2 have a similar radiological appearance to air trapping alterations in COPD on CT examination, a post-processing software, regularly employed in chronic lung disease, may help to stratify the severity of patients affected by SARS-CoV-2 [12–18].

This study aims to evaluate an artificial intelligence-based (AI) software, validated for the quantitative radiological COPD setting, for the semi-automated segmentation and volumetric lung quantification on chest CT, to assess lung involvement in SARS-CoV-2-related pneumonia, along with clinical and functional parameters.

## Materials and methods

Local Ethical Committee’s review of the protocol deemed that formal approval was not required owing to the retrospective, observational, and anonymous nature of this study.

### Patient population

From March 1, 2020, until April 10, 2020, we retrospectively enrolled all hospitalized patients with SARS-CoV-2 infection confirmed through a pan-coronavirus conventional polymerase chain reaction (PCR) assay with the following inclusion criteria: (1) RT-PCR-confirmed diagnosis of SARS-CoV-2 infection, (2) unenhanced chest CT, (3) complete laboratory test performed the same day of CT, (4) arterial blood gas (ABG) test performed on the same day of CT. The exclusion criteria were: (1) presence of severe breathing-induced artifacts on CT scan; (2) barotrauma (e.g., *pneumothorax*, pneumomediastinum, pneumopericardium, and soft tissue emphysema).

Patients were extracted from the electronic database of three different regional hospitals and referral center in Lombardy, a northern Italian Region (center 1: Monza San Gerardo Hospital, center 2: Vimercate Hospital, center 3: Desio Hospital).

All patients were hospitalized for mild-to-severe cases of pneumonia either in the intensive-care unit or in clinical wards, and the onset of symptoms was also reported.

### Clinical and laboratory data

For each patient, we recorded the following laboratory test results: (1) white blood cell differential including white blood cell count (WBC), neutrophil, lymphocyte, and platelet counts, (2) C-reactive protein (CRP) value, (3) arterial blood gas (ABG) test including arterial partial pressure of oxygen (PaO_2_) and arterial partial pressure of carbon dioxide (PaCO_2_), pH, bicarbonates (HCO_3_^−^), and PaO_2_/FiO_2_ (*P*/*F*) ratio.

Clinical data, including non-invasive ventilation (NIV) by continuous positive airway pressure (CPAP), invasive ventilation by endotracheal tube (ET), and outcome, were recorded, as well. No data regarding comorbidities were available.

### CT protocol

Unenhanced CT images were acquired with the patient in the supine position at full inspiration.

In the center 1 CT examinations, were performed using a 256-slice scanner (iCT Elite, Philips Healthcare) with 100 kV, automated mAs, thickness 2 mm, and increment 1 mm; in the center 2 using a 128-slice scanner (Revolution, General Electric) with 120 kV, automated mAs, thickness 2 mm, and increment 1 mm; and in the center 3 using a 16-slice scanner (Toshiba Aquilion RXL, Canon Medical Systems) with 120 kV, automated mAs, thickness 2 mm, and increment 1 mm.

Images were reconstructed with model-based iterative reconstruction algorithm (IMR, Philips Healthcare), hybrid iterative reconstruction algorithm (ASIR, GE Healthcare), and filtered back projection (FBP, Canon Medical System), respectively.

For each patient, the radiation dose, expressed as DLP (mGy cm), CTDI (mGy), and ED (mSv), was recorded.

### Quantitative image analysis

All CT images were analyzed quantitatively using a lung analysis validated software dedicated to COPD [12, 14–17] (IntelliSpace Portal, Philips Healthcare). The software uses the entire 3D CT volumes as input and outputs a probability map that indicates how likely voxels belong to a specific lung region. Proximal vasculature and bronchi were automatically removed.

Borders of each lung, fissures, and central airways were detected automatically. The tracheobronchial tree up to the subsegmental level was identified. Next, both lungs were differentiated from the surrounding chest wall and mediastinal structures. Lobe segmentation was then performed to allow automated delineation for each of the 5 pulmonary lobes using automatic lobe segmentation algorithm [16].

**Fig. 1:**
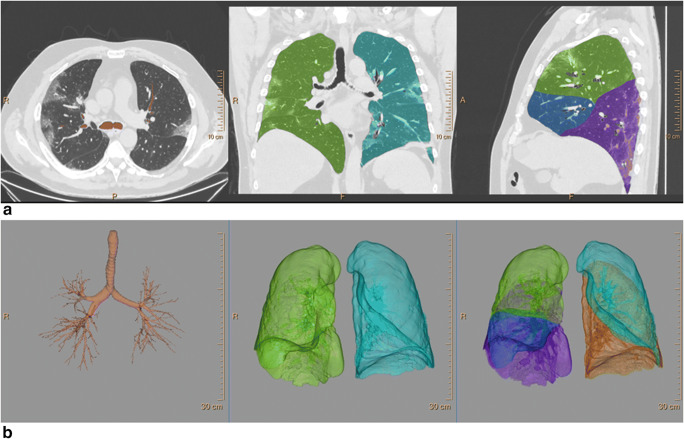
Automatic lobe segmentation process, derived from native unenhanced CT images in axial, coronal, and sagittal planes (**a** right, middle, and left panel, respectively), and the sequential segmentation of airways, lungs, and lobes (**b** right, middle, and left panel, respectively) in a 65-year-old man with SARS-CoV-2-related pneumonia

When lobe segmentation was completed, axial, sagittal, coronal, and volume-rendered images were displayed. A colored mask was superimposed onto the CT images using different colors for each lobe. By scrolling through the multiplanar images, it was possible for the operator to evaluate if the automated lobar segmentation was adequate (Fig. 1).

Ventilated lung volume (VLV, defined as the quantity of lung tissue ≤ − 700 HU on inspiratory CT), as well as GGO volume (GGO-V, defined as the quantity of lung tissue − 700 < *x* ≤ − 300HU on inspiratory CT), and consolidation volume (CONS-V, defined as the quantity of lung tissue > − 300 HU on inspiratory CT) were defined and computed both as absolute volume and as a percentage of total lung volume (TLV). The software distinguished automatically different lung alterations based on the absolute attenuation values, in particular GGOs and consolidations, mainly due to the presence of inflammatory cells, alveolar edema, and alveolar collapse.

Finally, we calculated the ratio between CONS-V, GGO-V, VLV (CONS-V/VLV and GGO-V/VLV, respectively), TLV (CONS-V/TLV, GGO-V/TLV, and GGO-V + CONS-V/TLV respectively), and the ratio between VLV and TLV (VLV/TLV).

### Qualitative image analysis

A senior radiologist, with at least 15 years of experience in chest imaging, and a radiologist in training with 4 years of experience reviewed CT images in the picture archiving and communication systems (PACS, Enterprise Imaging, AGFA Healthcare). Both lungs as well as each lung lobe were automatically segmented by software with manual edits by radiologist in training under senior radiologist’s supervision if needed.

For each CT exam, both the senior radiologist and the radiologist in training calculated the CT score proposed by Huang et al [18], according to the extent of GGO involvement in each lobe, as follows: “0” denoted no involvement, “1” less than 5% involvement, “2” 5–25%, “3” 26–49%, “4” 50–75%, and “5” more than 75%. Moreover, CT score was increased by one point in the presence of a crazy-paving pattern and by two points in case of consolidation. Therefore, a maximum CT score of 7 was possible for each lobe. The total CT score was defined as the sum of the scores for each of the five lobes and ranged from 0 to 35.

The evaluation of lesion density was visually performed based on the proportion of major SARS-CoV-2 CT findings, ground-glass opacities, and consolidations which were judged according to the international nomenclature defined by the Fleischner Society glossary [19] and peer-reviewed literature on viral pneumonia.

### Statistical analysis

Continuous variables were expressed as mean and standard deviations and, after assessing the normality distribution by using Kolmogorov-Smirnov test, were compared by using the Student *t* test. Categorical variables were expressed as median values and interquartile range (IQR) and compared by using the χ^2^ test or Fisher exact test, as appropriate. Correlations were computed with the Pearson or Spearman correlation coefficients. To assess agreement of CT score between the senior radiologist and the radiologist in training, we calculated Cohen’s kappa values (*κ*): kappa value equal or minor than 0 indicates no agreement while kappa value equal to 1 indicates perfect agreement. All tests were two-sided, and *p* < 0.05 was considered statistically significant. All statistical analyses were performed by using the SPSS statistical package software (version 26.0; SPSS).

## Results

### Demographic, clinical, and laboratory data of the entire cohort

**Fig. 2:**
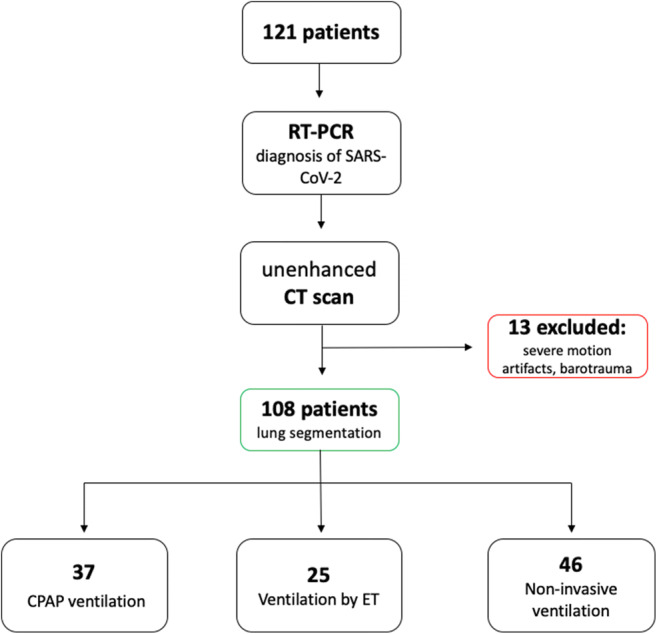
Flow chart of the study

A total of 121 patients were enrolled (*n* = 82 from center 1, *n* = 24 from center 2, and *n* = 15 from center 3); 13 were excluded due to the presence of motion artifacts or barotrauma. Flow chart in Fig. 2 summarizes the study design.

The final cohort was composed of 108 patients, the majority was male (*n* = 84, 77.8%) with a mean age of 63 years (± 11.4). Laboratory data of the entire cohort are summarized in Table 1. The diagnosis of SARS-CoV-2 infection was confirmed through a pan-coronavirus conventional polymerase chain reaction (PCR) assay.

**Table 1:** Clinical and laboratory data of the entire cohort

*N* = 108	
Age (years old ± SD)	68 ± 11.4
Sex male (*n*; %)	84 (77.8)
WBC (× 10^3^/mm^3^ ± SD)	8.22 ± 4.63
Neutrophils (× 10^3^/mm^3^ ± SD)	5.71 ± 3.08
Lymphocytes (× 10^3^/mm^3^ ± SD)	1.37 ± 1.35
PLT (× 10^3^/mm^3^ ± SD)	239.85 ± 120.48
CRP (mg/l ± SD)	10.25 ± 11.84
SaO_2_ (%, median, range)	95 (89–99)
PaO_2_ (mmHg ± SD)	90.79 ± 41.68
PaCO_2_ (mmHg ± SD)	35.75 ± 7.01
pH (± SD)	7.42 ± 0.30
HCO_3_^−^ (mEq/l ± SD)	25.43 ± 4.35
*P*/*F* ratio ± SD	234.09 ± 101.61

A total of 37 patients (34.2%) underwent CPAP ventilation, 25 (23.2%) invasive ventilation by ET, and 46 (42.6%) non-invasive ventilation.

At the end of the study, 18 patients (16.7%) were still hospitalized, 18 (16.7%) died, and 72 (66.6%) were discharged within 5 days. The median time of hospitalization patients was 19 days (IQR 9–40).

The mean timespan from symptom onset to CT examination was 13.1 (± 7.1) days.

### Software diagnostic performance

The operator’s interaction with the analysis procedure was minimized as much as possible. However, only in one case, lobar limits were corrected with a minor adjustment for interlobar boundaries in multiplanar images. The mean processing time for automated lung segmentation was 105 ± 13.4 s.

After setting the relative cutoff lung alteration values, the mean time for the analysis from lung segmentation to GGOs or consolidation quantification was 2.5 ± 1.4 s.

No manually input was needed for the GGO boundary assessment and automated quantification. In 22% of cases with lung consolidations, a manual input was needed to better define the boundary of lung alterations with a mean processing time of 183 ± 39 s.

### CT score, lung volumes, and radiation dose exposure

According to the *RSNA chest CT classification system for reporting COVID-19* [20], a total of 84 patients (78%) presented *typical CT imaging* features, while 24 (22%) *indeterminate* CT appearance. No *atypical* or *negative* appearance of CT features for SARS-CoV-2-related pneumonia has been found.

The median value of CT score was 24 (3–35), with no statistical difference between males and females (*p* = 0.857). The agreement between two readers was very good (*κ* = 0.91).

Overall, the mean TLV was 3861.78 ml (± 1241.72) and VLV 2701.48 ml (± 1337.59). The mean GGO-V was higher than CONS-V (860.02 ± 427.45 ml and 299.39 ± 318.49 ml, respectively). The ratios between GGO-V, CONS-V, TLV, and VLV were calculated and are summarized in Table 2.

**Table 2:** Lung volumes in the entire cohort

*N* = 108	Mean	SD	Range	Min	Max
VLV (ml)	2701.48	1337.56	6330	392	6722
TLV (ml)	3861.79	1241.72	6229	1316	7545
CONS-V (ml)	299.39	318.49	1368	0	1368
GGO-V (ml)	860.01	427.45	2332	176	2508
GGO-V/TLV (%)	23.98		65.00	2.79	67.80
CONS-V/TLV (%)	8.68		47.59	0	47.59
GGO-V/VLV (%)	49.62		373.79	3.25	377.04
CONS-V/VLV (%)	21.44		314.43	0	314.43
CONS-V + GGO-V/TLV (%)	32.66		80.23	5.86	86.09
VLV/TLV (%)	67.31		80.23	12.91	94.14

The overall radiation dose delivered to the patients had a mean DLP of 310 ± 128 mGy cm, mean of CTDI of 7.5 ± 3.1 mGy, and a mean ED of 5.3 ± 2.1 mSv.

### GGO volumes and laboratory data

**Fig. 3:**
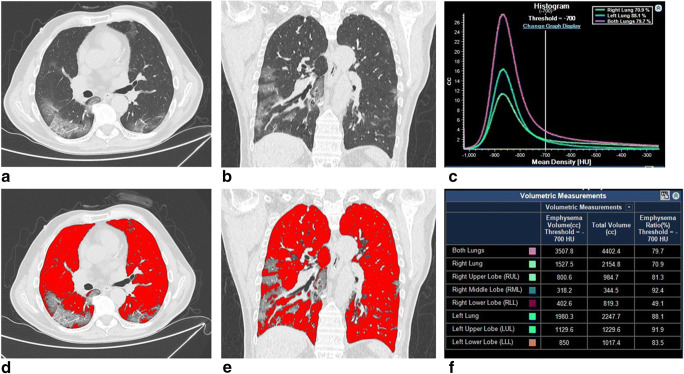
A 61-year-old man with SARS-CoV-2-related pneumonia. CT images in axial (**a**) and coronal (**b**) planes, with window width and level for the evaluation of the lung parenchyma. The images show the presence of bilateral ground-glass opacities and partial crazy-paving pattern. Final CT score: 12. Post-processed CT images in axial (**d**) and coronal (**e**) sections showing low attenuation areas based on thresholds of − 700 HU that correspond to ventilated lung volume (VLV). CT density histogram based on the attenuation lung analysis showing both long volumes that correspond to TLV (purple line), left (blue line), and right (green line) lung volumes (**c**). Post-processing volumes of different lung segments calculated by the software (**f**)

A positive correlation was found between GGO-V and WBC (r = 0.386, *p* < 0.001), neutrophils (*r* = 0.410, *p* < 0.001), platelets (*r* = 0.232, *p* = 0.008), PaCO_2_ (*r* = 0.205, *p* = 0.018), and HCO_3_^−^ values (*r* = 0.187, *p* = 0.034), while a negative correlation was obtained with SaO_2_ (*r* = − 0.240, *p* = 0.014) and *P*/*F* ratio (*r* = − 0.372, *p* < 0.001) values. GGO-V/TLV correlated positively with WBC (*r* = 0.369, *p* < 0.001), neutrophils (*r* = 0.446, *p* < 0.001), platelets (*r* = 0.182, *p* = 0.030), CRP (*r* = 0.190, *p* = 0.025), PaCO_2_ (*r* = 0.176, *p* = 0.036), and HCO_3_^−^ (*r* = 0.284, *p* = 0.002) values, and negatively with *P*/*F* (*r* = − 0.344, *p* < 0.001) value. GGO-V/VLV correlated positively with WBC (*r* = 0.384, *p* < 0.001), neutrophils (*r* = 0.454, *p* < 0.001), platelets (*r* = 0.190, *p* = 0.024), CRP (*r* = 0.233, *p* = 0.008), PaCO_2_ (*r* = 0.200, *p* = 0.020), and HCO_3_^−^ (*r* = 0.326, *p* = 0.001) values, and negatively with *P*/*F* ratio (*r* = − 0.380, *p* < 0.001) value (Fig. 3). All correlations between GGO volumes and other laboratory data values are summarized in Table 3.

**Table 3:** Correlations between lung volumes, laboratory data, and CT score. Statically significant correlations are highlighted in italic

		GGO-V	GGO-V/TLV	GGO-V/VLV	CONS-V	CONS-V/TLV	CONS-V/VLV	CONS-V + GGO-V/TLV	CT score
WBC	*r*	*0.386*	*0.369*	*0.384*	*0.299*	*0.294*	*0.299*	*0.375*	*0.322*
	*p*	< 0.001	< 0.001	< 0.001	0.001	0.001	0.001	< 0.001	< 0.001
Neutrophils	*r*	*0.410*	*0.446*	*0.454*	*0.289*	*0.300*	*0.312*	*0.439*	*0.378*
	*p*	< 0.001	< 0.001	< 0.001	0.002	0.001	0.001	< 0.001	< 0.001
Lymphocytes	*r*	0.074	0.055	− 0.045	*− 0.212*	*− 0.225*	*− 0.204*	− 0.106	− 0.020
	*p*	0.235	0.295	0.331	0.018	0.013	0.022	0.150	0.423
PLT	*r*	*0.232*	*0.182*	*0.190*	0.148	0.145	0.149	*0.177*	*0.233*
	*p*	0.008	0.030	0.024	0.064	0.067	0.061	0.033	0.010
CRP	*r*	0.042	*0.190*	*0.233*	*0.252*	*0.306*	*0.285*	*0.266*	*0.163*
	*p*	0.333	0.025	0.008	0.004	0.001	0.001	0.003	0.046
PaO_2_	r	− 0.070	− 0.009	0.005	0.093	0.106	0.086	0.038	− 0.027
	*p*	0.240	0.464	0.481	0.173	0.140	0.191	0.348	0.392
PaCO_2_	*r*	*0.205*	*0.176*	*0.200*	*0.229*	*0.227*	*0.227*	*0.208*	*0.264*
	*p*	0.018	0.036	0.020	0.009	0.010	0.010	0.017	0.003
SaO_2_	*r*	*− 0.240*	− 0.145	− 0.136	− 0.099	− 0.043	− 0.069	− 0.113	*− 0.199*
	*p*	0.014	0.095	0.128	0.185	0.350	0.269	0.155	0.035
pH	*r*	− 0.039	0.021	0.051	0.149	*0.162*	*0.149*	0.089	0.065
	*p*	0.349	0.417	0.307	0.068	0.048	0.034	0.186	0.257
HCO_3_^−^	*r*	*0.187*	*0.284*	*0.326*	*0.344*	*0.394*	*0.383*	*0.357*	*0.352*
	*p*	0.034	0.002	0.001	< 0.001	< 0.001	< 0.001	< 0.001	< 0.001
*P*/*F* ratio	*r*	*− 0.372*	*− 0.344*	*− 0.380*	*− 0.468*	*− 0.419*	*− 0.411*	*− 0.410*	*− 0.464*
	*p*	< 0.001	< 0.001	< 0.001	< 0.001	< 0.001	< 0.001	< 0.001	< 0.001
CT score	*r*	*0.735*	*0.810*	*0.840*	*0.725*	*0.717*	*0.742*	*0.847*	-
	*p*	< 0.001	< 0.001	< 0.001	< 0.001	< 0.001	< 0.001	< 0.001	-

### Consolidation volumes and laboratory data

A positive correlation was found between CONS-V and WBC (*r* = 0.299, *p* = 0.001), neutrophils (*r* = 0.289, *p* = 0.002), CRP (*r* = 0.252, *p* = 0.004), PaCO_2_ (*r* = 0.229, *p* = 0.009), and HCO_3_^−^ (*r* = 0.344, *p* < 0.001) values, while negative correlations with lymphocytes (*r* = − 0.212, *p* = 0.018) and *P*/*F* ratio (*r* = − 0.468, *p* < 0.001) values were obtained.

**Fig. 4:**
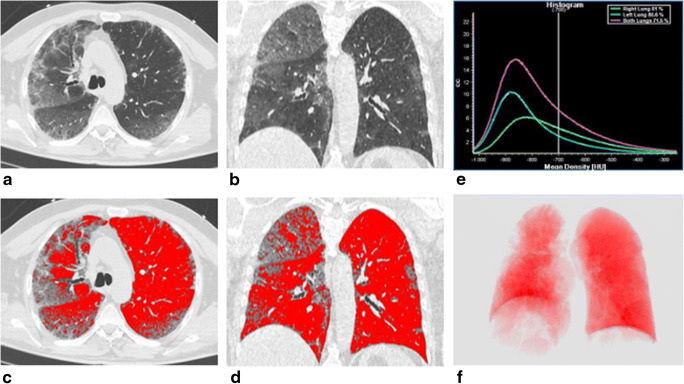
A 63-year-old man with SARS-CoV-2-related pneumonia. CT images in axial (**a**) and coronal (**b**) planes, showing the presence of bilateral and peripheral ground-glass opacities associated with septa thickening and crazy-paving pattern, bronchiectasis, and band thickening. Final CT score: 18. Post-processed CT images in axial (**c**), coronal (**d**) sections, and 3D reconstruction displaying the ventilated lung volume, characterized by attenuation value lower than − 700 HU (**f**). The density histogram (**e**) summarizes the distribution of lung parenchymal density of total lung volume (purple line), left lung volume (green line), and right lung volume (blue line)

CONS-V/TLV correlated positively with WBC (*r* = 0.294, *p* = 0.001), neutrophils (*r* = 0.300, *p* = 0.001), CRP (*r* = 0.306, *p* = 0.001), PaCO_2_ (*r* = 0.227, *p* = 0.010), pH (*r* = 0.162, *p* = 0.048), and HCO_3_^−^ (*r* = 0.394, *p* < 0.001) values, while negative correlations with lymphocytes (*r* = − 0.225, *p* = 0.013) and *P*/*F* ratio (*r* = − 0.419, *p* < 0.001) values were found (Fig. 4).

CONS-V/VLV correlated positively with WBC (*r* = 0.299, *p* = 0.001), neutrophils (*r* = 0.312, *p* = 0.001), CRP (*r* = 0.285, *p* = 0.001), PaCO_2_ (*r* = 0.227, *p* = 0.010), and HCO_3_^−^ (*r* = 0.383, *p* < 0.001) values, while negative correlations with lymphocytes (*r* = − 0.204, *p* = 0.022) and *P*/*F* ratio (*r* = − 0.411, *p* < 0.001) values were found.

All correlations between GGO volumes and other laboratory data are summarized in Table 3.

### CT score, lung volumes, and laboratory data

**Fig. 5:**
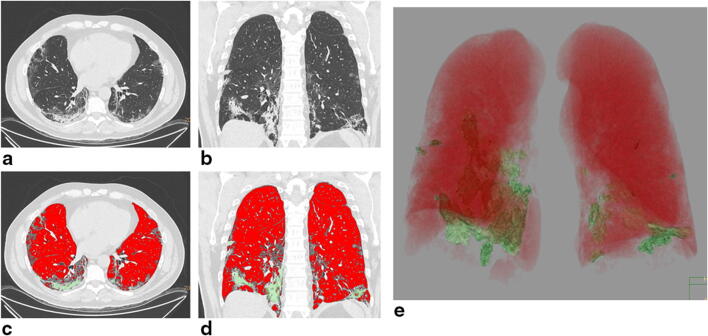
A 67-year-old man with SARS-CoV-2 related pneumonia. CT images in axial (**a**) and coronal (**b**) planes, showing bilateral multiple lobular ground-glass opacities associated with consolidations especially in the lower lobes. Final CT score: 24. Post-processed CT images in axial (**c**), coronal (**d**) sections and 3D reconstruction showing the ventilated lung volume (red colored), with an attenuation value lower than − 700 HU, and consolidation volumes (green colored) (**e**). The remaining volume is referred to as ground-glass opacities volume with a density between − 700 HU and − 350 HU

Positive correlations between CT score and WBC (*r* = 0.322, *p* < 0.001), neutrophils (*r* = 0.378, *p* < 0.001), platelets (*r* = 0.233, *p* = 0.010), CRP (*r* = 0.163, *p* = 0.046), PaCO_2_ (*r* = 0.264, *p* = 0.003), and HCO_3_^−^ (*r* = 0.352, *p* < 0.001) values were found, while negative correlations with SaO_2_ (*r* = − 0.199, *p* = 0.035) and *P*/*F* (*r* = − 0.464, *p* < 0.001) were obtained (Fig. 5).

Strong positive correlations between CT score and all lung volumes were found (all *r* > 0.5 and all *p* < 0.001). All correlations are summarized in Table 3.

### Outcome

SaO_2_ values were significantly lower in patients who underwent CPAP ventilation (*p* = 0.037).

WBC, neutrophils, platelets, PaCO_2_, HCO_3_^−^, and *P*/*F* values showed a statistically significant difference in patients who underwent invasive ventilation by ET compared with other patients treated with non-invasive ventilation techniques (all *p* > 0.05). All differences between groups are reported in Table 4.

**Table 4:** Laboratory data, lung volumes, and CT score in patients underwent CPAP and invasive ventilation by ET. Statically significant differences are highlighted in italic

*N* = 108	CPAP			ET		
	Yes	No	*p* value	Yes	No	*p* value
Age (years old ± SD)	66 ± 11	63 ± 10	0.339	63 ± 7	65 ± 11	0.674
Sex male (*n*; %)	29; 26.85	42; 38.88	0.454	22; 20.37	49; 45.37	0.088
WBC (× 10^3^/mm^3^ ± SD)	7.35 ± 3.30	8.75 ± 5.54	0.286	10.72 ± 3.55	7.26 ± 3.55	*0.002*
Neutrophils (× 10^3^/mm^3^ ± SD)	5.49 ± 2.76	5.81 ± 3.35	0.764	7.36 ± 3.44	5.18 ± 2.83	*0.005*
Lymphocytes (× 10^3^/mm^3^ ± SD)	1.26 ± 0.91	1.47 ± 1.78	0.815	1.30 ± 0.86	1.40 ± 1.59	0.708
PLT (× 10^3^/mm^3^ ± SD)	222.54 ± 111.56	246.50 ± 115.36	0.325	270.72 ± 113.88	224.56 ± 112.14	*0.062*
CRP (mg/l ± SD)	7.47 ± 6.99	7.88 ± 8.64	0.953	9.87 ± 11.37	6.93 ± 6.25	0.768
SaO_2_ (%, median, range)	93 (89–94)	95 (92–99)	*0.037*	95 (91–98)	94 (91–96)	0.081
PaO_2_ (mmHg ± SD)	40.34 ± 96.64	85.48 ± 26.46	0.507	87.62 ± 23.33	91.01 ± 36.32	0.436
PaCO_2_ (mmHg ± SD)	38.85 ± 5.67	37.24 ± 7.82	0.266	39.91 ± 10.05	34.85 ± 5.67	*0.029*
pH (± SD)	7.44 ± 0.05	7.38 ± 0.42	0.769	7.42 ± 0.37	7.40 ± 0.55	0.384
HCO_3_^−^ (mEq/l ± SD)	24.40 ± 4.12	26.30 ± 4.31	0.383	27.80 ± 4.95	24.59 ± 3.70	*0.010*
*P*/*F* ratio ± SD	206.97 ± 84.67	247.39 ± 110.12	0.080	185.63 ± 92.28	248.12 ± 100.88	*0.007*
VLV (ml, mean ± SD)	2524.16 ± 1276.04	2795.57 ± 1294.35	0.245	2326.00 ± 1076.47	2820.52 ± 1338.98	0.176
TLV (ml, mean ± SD)	3834.72 ± 1219.99	3894.16 ± 1216.32	0.672	3882.68 ± 1118.17	3865.67 ± 1251.95	0.815
CONS-V (ml, mean ± SD)	337.54 ± 267.01	292.67 ± 350.59	0.057	472.32 ± 411.29	251.10 ± 256.77	*0.024*
GGO-V (ml, mean ± SD)	972.83 ± 466.38	804.25 ± 385.32	0.107	1085.36 ± 376.29	792.63 ± 417.26	*< 0.001*
GGO-V/TLV (%, median, range)	22.10 (2.79–61.50)	26.97 (3.44–67.80)	0.160	28.81 (3.80–49.9)	18.97 (2.84–59.9)	*0.001*
CONS-V/TLV (%, median, range)	7.49 (3.12–38.8)	4.96 (0–35.5)	0.127	11.61 (11.54–27.48)	5.08 (16.54–47.59)	*0.033*
GGO-V/VLV (%, median, range)	31.40 (3.25–110.44)	28.72 (6.78–298.41)	0.172	48.52 (29.8–377.04)	26.24 (18.91–198.0)	*0.001*
CONS-V/VLV (%, median, range)	11.29 (1.12–22.43)	6.68 (0–11.54)	0.740	19.66 (31.41–314.43)	7.13 (1.11–198.31)	*0.041*
VLV/TLV (%, median, range)	70.48 (12.91–45.60)	74.38 (39.65–99.01)	0.132	59.04 (11.32–70.84)	75.82 (70.11–94.14)	*0.002*
CONS-V + GGO-V/TLV (%, median, range)	29.52 (5.86–44.30)	25.62 (7.98–54.31)	0.131	40.96 (38.53–86.09)	24.18 (17.67–30.01)	*0.001*
CT score (median, range)	27 (20–31)	22 (16–29)	0.370	30 (10–35)	21 (7–23)	*0.035*

No statistically significant differences between all pulmonary volumes and CPAP ventilation were found. Patients underwent invasive ventilation by ET showed significantly higher values of pathological lung volumes, expressed as CONS-V, GGO-V, GGO-V/TLV, CONS-V/TLV, GGO-V/VLV, CONS-V/VLV, and GGO-V + CONS-V/TLV (all *p* < 0.05). Moreover, patients underwent invasive ventilation by ET showed a median CT score value significantly higher (*p* = 0.035) (Table 4).

No significant differences between all pulmonary volumes and patients’ death were found (all *p* values > 0.05).

## Discussion

As the clinical spectrum of COVID-19 infection ranges from mild illness to ARDS with high mortality risk, an early clinical marker that helps in determining prognosis is needed, to correctly establish the appropriate management. In this scenario, chest CT has a major role both in the detection and in the characterization of SARS-CoV-2-related pneumonia, allowing a better stratification of illness severity and prompt therapeutic choices.

Recent studies highlighted that GGOs are present in all cases, with multilobe and posterior involvement in over 90% [20].

In this setting, we hypothesized that the evaluation of pathological lung volumes, represented by GGOs and consolidation (GGO-V and CONS-V, respectively), could be useful to perform an accurate staging of the disease and, more importantly, to establish management and determine prognosis, as recently investigated also by other groups in literature [21, 22].

Image post-processing software employed in our study, validated usefully in clinical and radiological quantitative COPD setting [12, 16, 17], represents a semi-automatic tool to visualize and measure total lung volume, residual lung volume, and ventilated parenchyma, and in particular, by modifying the cutoff level set between − 300 and − 700 HU to derive a specific windowing setting for SARS-CoV-2 patients, the algorithm was able to automatically identify GGOs. Our cutoff value was derived from several similar approach in literature [15], and specifically set also from the clinical everyday practice.

A recent paper from Lanza et al [15] tried to provide a quantitative analysis of SARS-CoV-2-related pneumonia through a dedicated automated software (3D slicer equipped with chest imaging protocol), and found in their series some cutoff points, in terms of percentage, of compromised lung volume related to the risk of need ventilation. These results strengthen the importance of quantitative method to assess the severity and triaging of patients with SARS-CoV-2-related pneumonia. Differently from our series, the authors did not differentiate in their analysis the ground-glass opacities from the consolidations.

More recently Feng et al [14] applied a deep learning-based software for quantification of COVID-19 lung involvement and compared the obtained results with those from a conventional visual CT scoring, highlighting the potential benefit for the estimation of disease severity.

To the best of our knowledge, this is the first study to categorize and quantify the two different main lung alterations, both SARS-CoV-2-related GGOs and consolidations, through application of semi-automated software, correlating the quantitative results with the final outcome of patients.

Our results showed that SARS-CoV-2-related pneumonia primarily manifests as diffuse and bilateral GGOs, confirmed by higher GGO-V values in comparison with CONS-V, in line with previous studies [23–25] (24% vs 9%), and the amount of both lung findings (CONS-V and GGO-V) are quantitatively higher in patients who undergo invasive ventilation by ET in comparison with NIV (41% vs 29%).

We found a strong correlation between GGO-V, considered alone and as a ratio to TLV and VLV, and different laboratory data, in particular WBC (*p* = 0.002), neutrophils (*p* = 0.005), platelet counts (*p* = 0.062), PaCO_2_ (*p* = 0.029), and bicarbonates (*p* = 0.010), suggesting that GGOs are linked to inflammatory status and the clinical outcome, including the need for mechanical ventilation. Interestingly, as the shortage of intensive care unit (ICU) beds and mechanical ventilators has been a major concern during the pandemic, the possibility to be able to predict the need for ICU admission may be crucial to allow a proper resource allocation and improve patient survival.

The same results can be appreciated by evaluating CONS-V, alone, and as a ratio to TLV and VLV. However, we found weaker correlations with laboratory data in comparison with GGO-V values, as they do not represent a typical pattern of SARS-CoV-2-related pneumonia [26].

Moreover, the evaluation of total pathological parenchyma, considered CONS-V + GGO-V/TLV, allows to deeper understand the disease mechanisms, as it correlated with WBC, neutrophil and platelet counts, CRP, PaCO_2_, pH, and HCO_3_^−^ values, and could help clinicians in taking prompt clinical decisions, including early endotracheal intubation.

Interestingly, we observed a strong correlation between hypoxia, in terms of *P*/*F* ratio, hypercapnia, and GGO-V and CONS-V, which was also confirmed by the CT score: this finding adds relevance to the present study, as these indicators are used in clinical practice to shepherd the weaning from mechanical ventilation and directly correlate to the outcome.

To test software reliability, we calculated CT score for each examination, finally confirming its agreement with all lung volumes, in particular in terms of GGO-V, CONS-V/TLV, and GGO-V + CON-V/TLV: our results showed that the visual CT score strongly correlated with long volume analysis. However, the CT score proposed by Huang et al [18] is operator-dependent and time-consuming, while the software performs an automatic calculation that can give a more accurate staging in terms of pulmonary involvement. The merge of human and AI-driven results adds together, completing a global and accurate evaluation: the automatic model proposed is more precise in terms of quantification of consolidation and GGOs, but, on the other hand, human supervision is still necessary.

The fact that elaborations on chest CT may predict the needs of SARS-CoV-2 patients in terms of ventilatory support also highlights the objective of early chest CT examination in suspects for SARS-CoV-2 infection or ambiguous RT-PCR results.

Finally, we applied CT lung volumes to clinical practice, in particular, following patients during hospitalization, and we found that CT lung volumes, in particular residual lung volumes, were lower in patients with the need for endotracheal intubation.

Patients have undergone CPAP did not show any significant difference in terms of laboratory data and CT lung volumes while, on the other hand, patients undergone invasive ventilation by ET showed significantly different values in terms of laboratory data and CT lung volumes. As previously mentioned, all CT volumes significantly differed between the two groups, in particular GGO-V/TLV, GGO-V/VLV, and CONS-V + GGO-V/TLV, confirming that the total amount of pathologically involved lung parenchyma is higher in patients that need a more invasive ventilation approach.

The present study has some limitations. Firstly, chest CTs were retrospectively evaluated and not executed upon hospital admission but at different clinical stages. Secondly, the software was not specifically designed to assess SARS-CoV-2, and, although the automatic tool may simplify the work of radiologists, in some cases, it needs human supervision, especially in consolidative alterations presenting as irregular band, or close to pleural effusion, in a total of 24 patients from our series. Based on the results of the current study, further research should focus on the development of dedicated software to assess lung involvement in viral pneumonia. Finally, our results should be considered preliminary and not be used to drive clinical decisions, even if they carry an undeniable contribution which may drive further research, and should be considered also as a tool in the follow-up of SARS-COV-2 patients, to assess, through quantitative lung parenchyma evaluation, the response to treatment.

In conclusion, post-processing software in SARS-CoV-2 patients resulted as a reliable tool to obtain a quantification of lung involvement, which significantly correlated to laboratory data and, in particular, to patient’s outcomes, especially concerning invasive ventilation.

## Abbreviations

AI: Artificial intelligence
CONS-V: Consolidation volume
COPD: Chronic obstructive pulmonary disease
CRP: C-reactive protein
CT: Computed tomography
GGOs: Ground-glass opacities
GGO-V: Ground-glass opacity volume
HCO_3_^−^: Bicarbonates
HU: Hounsfield unit
PaCO_2_: Partial pressure of carbon dioxide
PaO_2_: Partial pressure of oxygen
pH: Arterial pH
PLT: Platelets
RT-PCR: Reverse-transcription polymerase chain reaction
TLV: Total lung volume
VLV: Ventilated lung volume
WBC: White blood cells

## References

[1] Xu X, Yu C, Qu J et al (2020) Imaging and clinical features of patients with 2019 novel coronavirus SARS-CoV-2. Eur J Nucl Med Mol Imaging 47(5):1275–1280 10.1007/s00259-020-04735-9 PMC7080117 32107577

[2] Use of laboratory methods for SARS diagnosis. In: who.int. https://www.who.int/csr/sars/labmethods/en/. Accessed 10 July 2020

[3] Chan JF-W, Yip CC-Y, To KK-W et al (2020) Improved molecular diagnosis of COVID-19 by the novel, highly sensitive and specific COVID-19-RdRp/Hel real-time reverse transcription-PCR assay validated *in vitro* and with clinical specimens. J Clin Microbiol 58(5):e00310-20 10.1128/JCM.00310-20 PMC7180250 32132196

[4] Li B, Li X, Wang Y et al (2020) Diagnostic value and key features of computed tomography in coronavirus disease 2019. Emerg Microbes Infect 9(1):787–793 10.1080/22221751.2020.1750307 PMC7191895 32241244

[5] Xie J, Tong Z, Guan X, Du B, Qiu H, Slutsky AS (2020). Critical care crisis and some recommendations during the COVID-19 epidemic in China. Intensive Care Med.

[6] Zach JA, Newell JD, Schroeder J et al (2012) Quantitative computed tomography of the lungs and airways in healthy nonsmoking adults. Invest Radiol 47(10):596–602 10.1097/RLI.0b013e318262292e PMC3703944 22836310

[7] Newell JD, Sieren J, Hoffman EA (2013). Development of quantitative computed tomography lung protocols. J Thorac Imaging.

[8] Washko G (2010). Diagnostic Imaging in COPD. Semin Respir Crit Care Med.

[9] Silva M, Milanese G, Seletti V, Ariani A, Sverzellati N (2018). Pulmonary quantitative CT imaging in focal and diffuse disease: current research and clinical applications. Br J Radiol.

[10] Mascalchi M, Camiciottoli G, Diciotti S (2017). Lung densitometry: why, how and when. J Thorac Dis.

[11] den Harder AM, de Boer E, Lagerweij SJ et al (2018) Emphysema quantification using chest CT: influence of radiation dose reduction and reconstruction technique. Eur Radiol Exp 2(1):30 10.1186/s41747-018-0064-3 PMC6220000 30402740

[12] de Boer E, Nijholt IM, Jansen S et al (2019) Optimization of pulmonary emphysema quantification on CT scans of COPD patients using hybrid iterative and post-processing techniques: correlation with pulmonary function tests. Insights Imaging 10(1):102. 10.1186/s13244-019-0776-9 10.1186/s13244-019-0776-9 PMC6779684 31591646

[13] Fischer AM, Varga-Szemes A, Martin SS et al (2020) Artificial intelligence-based fully automated per lobe segmentation and emphysema-quantification based on chest computed tomography compared with global initiative for chronic obstructive lung disease severity of smokers. J Thorac Imaging 35:S28–S34 10.1097/RTI.0000000000000500 32235188

[14] Feng P, Lin L, Bo L et al (2020) A novel deep learning-based quantification of serial chest computed tomography in coronavirus disease 2019 (COVID-19). 10.21203/rs.3.rs-38083/v1 10.1038/s41598-020-80261-w PMC7801482 33432072

[15] Lanza E, Muglia R, Bolengo I et al (2020) Quantitative chest CT analysis in COVID-19 to predict the need for oxygenation support and intubation. Eur Radiol. 10.1007/s00330-020-07013-2 10.1007/s00330-020-07013-2 PMC7317888 32591888

[16] Bae K, Jeon KN, Lee SJ (2016). Severity of pulmonary emphysema and lung cancer: analysis using quantitative lobar emphysema scoring. Medicine (Baltimore).

[17] Lim H, Weinheimer O, Wielpütz MO (2016). Fully automated pulmonary lobar segmentation: influence of different prototype software programs onto quantitative evaluation of chronic obstructive lung disease. PLoS One.

[18] Huang G, Gong T, Wang G et al (2020) Timely diagnosis and treatment shortens the time to resolution of coronavirus disease (COVID-19) pneumonia and lowers the highest and last CT scores from sequential chest CT. AJR Am J Roentgenol 30:1–7 10.2214/AJR.20.23078 32223665

[19] Hansell DM, Bankier AA, MacMahon H (2008). Fleischner society: glossary of terms for thoracic imaging. Radiology.

[20] Sun Z, Zhang N, Li Y, Xu X (2020). A systematic review of chest imaging findings in COVID-19. Quant Imaging Med Surg.

[21] Belfiore MP, Urraro F, Grassi R et al (2020) Artificial intelligence to codify lung CT in Covid-19 patients. Radiol Med 125(5):500–504 10.1007/s11547-020-01195-x PMC7197034 32367319

[22] Colombi D, Bodini FC, Petrini M et al (2020) Well-aerated lung on admitting chest CT to predict adverse outcome in COVID-19 pneumonia. Radiology. 17:201433 10.1148/radiol.2020201433 PMC7233411 32301647

[23] Singhal T (2020). A review of coronavirus disease-2019 (COVID-19). Indian J Pediatr.

[24] Cheng Z, Lu Y, Cao Q et al (2020) Clinical features and chest CT manifestations of coronavirus disease 2019 (COVID-19) in a single-center study in Shanghai, China. AJR Am J Roentgenol 14:1–6 10.2214/AJR.20.22959 32174128

[25] Zhou S, Wang Y, Zhu T, Xia L (2020). CT features of coronavirus disease 2019 (COVID-19) pneumonia in 62 patients in Wuhan, China. AJR Am J Roentgenol.

[26] Frater JL, Zini G, d’Onofrio G, Rogers HJ (2020) COVID-19 and the clinical hematology laboratory. Int J Lab Hematol. 42(Suppl 1):11–18. 10.1111/ijlh.13229 10.1111/ijlh.13229 PMC7264622 32311826

